# Acute Pediatric Pancreatitis Presenting with Splenic Vein Thrombosis

**DOI:** 10.7759/cureus.29462

**Published:** 2022-09-22

**Authors:** Julia Villanueva, Martha Chavez, La Nyka A Christian, Louisdon Pierre, Aziza Sedrak

**Affiliations:** 1 Pediatric Critical Care, The Brooklyn Hospital Center, St. George's University School of Medicine, Brooklyn, USA; 2 Internal Medicine, The Brooklyn Hospital Center, St. George's University School of Medicine, Brooklyn, USA; 3 Pediatrics, The Brooklyn Hospital Center, Brooklyn, USA; 4 Pediatrics, Icahn School of Medicine at Mount Sinai, New York, USA; 5 Pediatric Critical Care, The Brooklyn Hospital Center, Brooklyn, USA; 6 Pediatric Hematologist, Oncologist, The Brooklyn Hospital Center, Brooklyn, USA

**Keywords:** vascular complications, splenic vein thrombosis, acute pancreatitis, surgery, gastroenterology, pediatrics

## Abstract

Acute pancreatitis among the pediatric population can result from genetic disorders, anatomic anomalies, gallstones, trauma, and medications; trauma and idiopathic causes being the most common. Although chronic pancreatitis presents with increased severe long-term complications, acute pancreatitis presents with its share of complications such as fistulas, pseudocysts, and venous abnormalities. With an increase in hospitalization rates of acute pancreatitis among the pediatric population, the importance of understanding rare complications and how to further recognize these complications can aid in the diagnosis, medical management, and intervention necessary to optimize a patient's outcome. Our patient presented with a rare complication of splenic vein thrombosis (SVT), which is a complication that can also be observed in adults with acute pancreatitis. SVTs are uncommon in both the adult and pediatric populations, and they have received little attention or research in the pediatric population. We report a case that will highlight a rare case of SVT presenting in a pediatric patient with acute necrotizing gallstone pancreatitis.

## Introduction

Pancreatitis is seen in both adult and pediatric populations. However, acute pancreatitis is considered to be infrequent in individuals under the age of 20 [1]. Over the past two decades, there have been several studies reporting on the increased number of cases of acute, recurrent, and chronic pancreatitis in this age group [2]. The clinical and etiological presentation of acute pancreatitis in the pediatric population tends to differ from that of the adult population, with the most common symptoms consisting of nausea, vomiting, and abdominal pain, all of which vary in duration [3]. Etiology tends to be multifactorial, with the most known etiologies consisting of medication adverse effects, trauma, infections, anatomic anomalies such as choledochal cysts, and abnormal union of the pancreatobiliary junction [4]. The International Study Group of Pediatric Pancreatitis (INSPPIRE) uses the following criteria to define and diagnose acute pediatric pancreatitis: At least two out of the three following conditions are required for acute pancreatitis to be diagnosed: acute abdominal pain, serum amylase, and/or lipase activity at least three times greater than the upper limit of normal, and imaging findings representing or hinting at possible acute pancreatitis [5].

Splenic vein thrombosis (SVT) as a pancreatitis complication is more common in people aged 30-50 years old [6,7], and it is most common in adults with chronic pancreatitis. Several complications associated with pancreatitis in the pediatric population include fistulas, pseudocysts, removal of pancreatic fluids via drainage, and vascular complications, with pancreatic pseudocysts being the most common [3]. Of these complications, the most reported complication has been the development of pseudocysts [3]. As the hospitalization and admission rates for acute pediatric pancreatitis increase [8,9], it is important for clinicians to recognize SVT as a potential complication of acute pancreatitis for appropriate management.

## Case presentation

**Figure 1:**
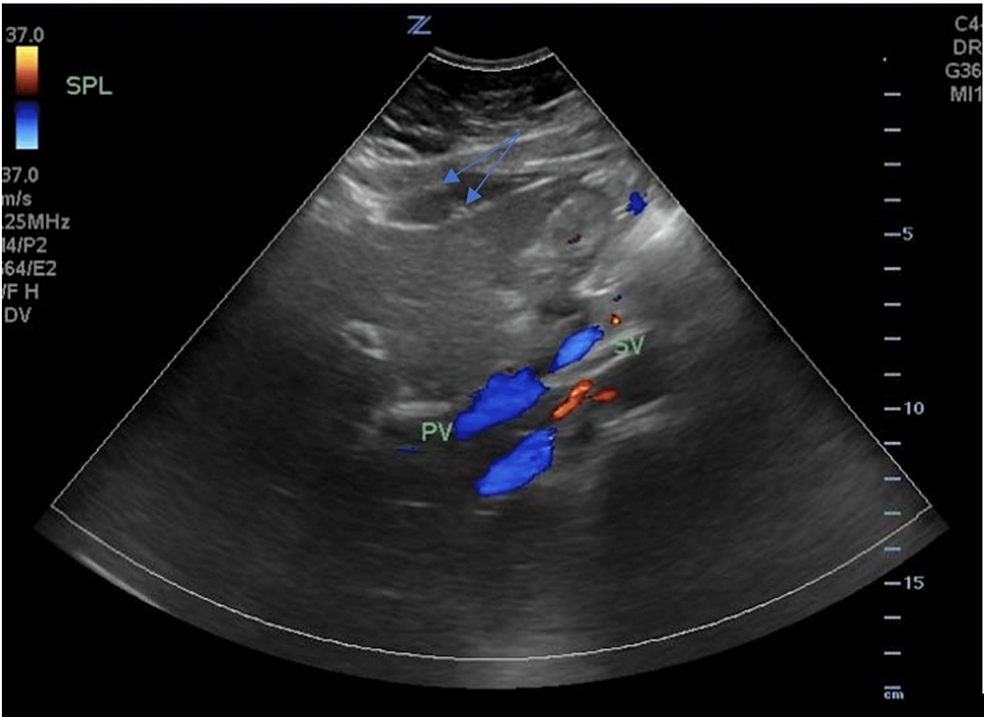
The ultrasound of the right upper quadrant of the abdomen shows multiple stones along the gallbladder without associated gallbladder wall thickening or pericholecystic fluid.

On presentation to the emergency department (ED), an 11-year-old African American female, obese, weighing 96 pounds with a height of four feet and five inches displayed intense generalized abdominal pain followed by intractable vomiting for one day. The clinical exam was remarkable for a soft abdomen with tenderness to palpation in the left upper quadrant and migration to the suprapubic and epigastric regions. No guarding or rebound tenderness was elicited. According to the lab reports, amylase was 1724 U/L, lipase was greater than 1200 U/L, triglycerides were 70 mg/dL, white blood count showed a left shift of 78.6% neutrophils, total bilirubin was 2.0 mg/dl, direct bilirubin was 1.1 mg/dl, aspartate aminotransferase was 178 U/L, and alanine aminotransferase was 78 U/L. The patient tested negative for coronavirus disease via both COVID-19 rapid and polymerase chain reaction (PCR) tests. An abdominal ultrasound revealed multiple stones layered along the dependent aspect of the gallbladder without associated gallbladder wall thickening or pericholecystic fluid (Figure 1).

A computed tomography (CT) scan of the abdomen and pelvis (A/P) with contrast revealed a diffusely enlarged pancreas with significant surrounding fat stranding and fluid tracked from the peripancreatic space throughout the upper abdomen into the pelvis. These findings were most compatible with acute pancreatitis. There was also decreased attenuation of the pancreas diffusely, likely secondary to edema, but early necrotizing pancreatitis could not be excluded at the time. No evidence of focal thrombi was noted on imaging at this time. Due to this imaging, a short-interval follow-up contrast-enhanced CT was recommended. The patient received an intravenous (IV) dose of one liter of normal saline, Zofran, famotidine, and morphine. Surgery was consulted and the patient was admitted to the floor.

**Figure 2:**
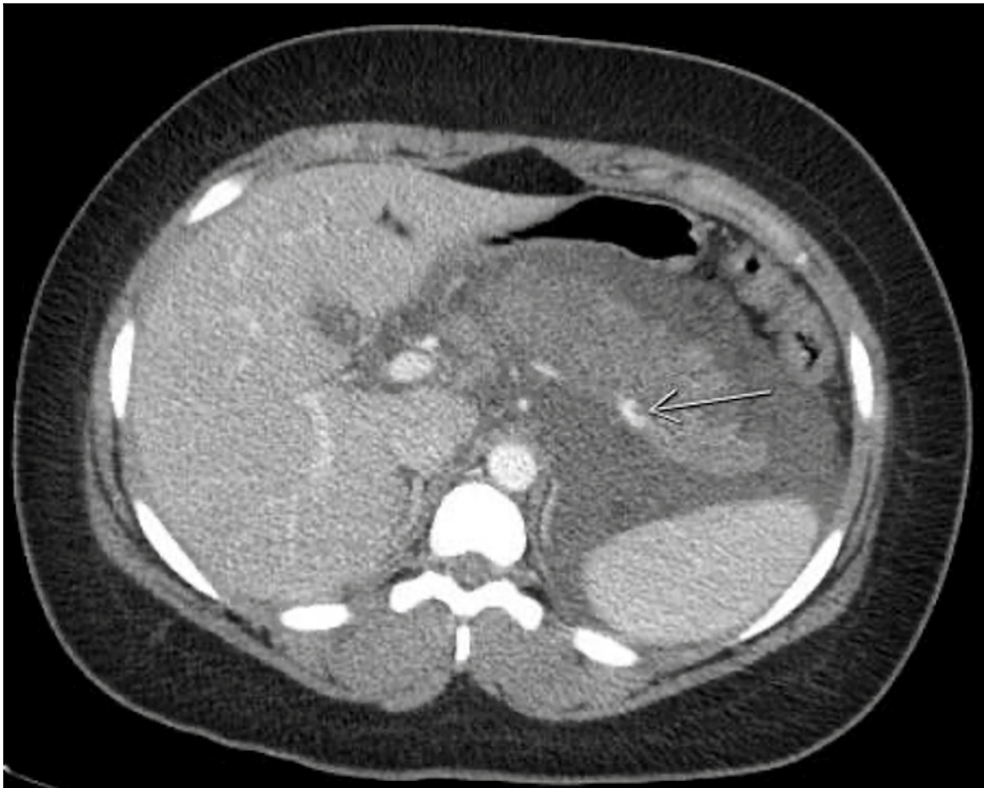
A repeat CT of the abdomen and pelvis (axial view) with contrast, performed two days after the first CT showed worsening pancreatitis with signs of liquefactive necrosis associated with left greater than right basilar subsegmental consolidation and showed interval narrowing of the splenic vein with nonocclusive thrombus.

However, on hospital day (HD) two, the patient spiked a fever of 102.5 degrees Fahrenheit, so she was started on IV Ceftriaxone and IV Metronidazole. She was escalated to IV meropenem later. The patient had a repeat CT A/P with contrast on HD two, which showed worsening pancreatitis with signs of liquefactive necrosis associated with left greater than right basilar subsegmental consolidation likely due to atelectasis. There was also interval narrowing of the splenic vein with two focal nonocclusive thrombi (Figure 2).

The patient was treated with therapeutic subcutaneous Lovenox at 1 mg/kg given the SVT finding and the absence of a central venous catheter, midline catheter, or peripherally inserted central catheter, causing a possible induced thrombus. Lovenox was dosed using the patient's ideal body weight and the dosage was titrated based on factor Xa levels. On HD seven, ecchymosis was noted on her foot, so hematology was consulted. It was recommended to perform a thrombophilia workup and continue the current dose of Lovenox.

Up until HD 11, the patient's labs were continuously monitored, and on HD 13, the patient’s Lovenox was transitioned to a heparin infusion for preparation of cholecystectomy and titrated with a repeat activated partial thromboplastin time (apTT) test every six hours. The patient reached a therapeutic range of 14 units/kg/hr for the patient’s ideal body weight. The heparin infusion was discontinued four hours before surgery on HD 15, and the cholecystectomy was uneventful. Postoperatively, the diet was gradually advanced, which the patient tolerated well. On HD 16, on restarting the patient on prophylactic anticoagulation, she was recommended subcutaneous Lovenox 40 mg once daily for one month. The patient was successfully discharged.

## Discussion

Pancreatitis is rare in the pediatric population [4], and it is rarely seen with vascular complications among all age groups [2]. There is limited evidence on the incidence, presentation, and complications associated with SVT seen in pediatric pancreatitis patients, making it challenging to evaluate and compare data thoroughly. For this reason, we will evaluate our patient based on a variety of reported studies and cases of SVT as a complication of acute pancreatitis among all age groups as a source of reference for the patient.

SVT prevalence has been reported to range from 1.2% to 20% in acute pancreatitis, with a pooled prevalence of 10.4% among reported studies [10]. Additionally, when looking at other SVT locations such as the portal vein and superior mesenteric vein, the prevalence was reported to be 6.2% and 2.7%, respectively, which emphasized a greater chance of having an SVT as a possible complication of acute pancreatitis [10]. Consequently, it can be assumed that with an increase in acute pancreatitis among the pediatric population, an increase in SVT as a complication of pancreatitis could occur as well [2].

The pathogenesis of SVT resulting from acute pancreatitis originates from a variety of causes, such as inflammatory changes accompanied by local pro-thrombotic factors, in which changes can be seen within the vascular endothelium [11,12]. Additionally, decreased pancreatic perfusion and pseudocysts can be seen in the acute progression of the disease and later pancreatic fibrosis [11,12]. This differs from other etiologies of SVT because it tends to be present for a shorter duration compared to malignancies and hypercoagulable states leading to SVT [11,12]. Given the patient’s body mass index (BMI) of 28 and the significant association between obesity and thrombotic disease [13], the patient’s risk for a hypercoagulable state was significantly elevated. Therefore, as part of the treatment and management course, hematology was involved during hospital admission and follow-up appointments to optimize management and treatment plans.

Patients with the following characteristics are suspected of having SVT: patients with a history of pancreatitis or gastrointestinal bleeding; patients with splenomegaly without portal vein hypertension; cirrhosis; or hematologic disease; and finally, patients with gastric varices alone [14]. SVT presents in most cases with gastric varices [15]. If there are gastric varices but no esophageal varices, or if gastric varices are more prominent than esophageal varices, then SVT should be suspected [15]. Patients with SVT can be asymptomatic or can present with a variety of symptoms. These symptoms include abdominal pain, variceal bleeding, splenomegaly, and thrombocytopenia [15]. In 60% of patients, pancreatitis is the initiating cause of thrombosis. However, the diagnosis of SVT in these patients does not always occur during an acute attack [14].

Many years ago, most cases of SVT were distinguished at postmortem autopsies[14]. With the increased development of medical imaging techniques such as celiac angiography and splenoportography, most cases are now easily detectable [14]. Since abdominal CT is used for patients with acute pancreatitis as well as preoperative patients with chronic pancreatitis, SVT is often an incidental finding in the CT scan [14]. While our patient initially received an abdominal ultrasound in the ED, which showed multiple stones along the gallbladder, a CT A/P with contrast was performed to rule out acute pancreatitis. CT imaging confirmed the diffusely enlarged pancreas with significant surrounding fat stranding and fluid, which is most compatible with acute pancreatitis. Furthermore, our patient followed the common trend where SVT was detected by a CT scan performed for other reasons, which in this case was acute pancreatitis. The CT scan showed two focal nonocclusive thrombi in the splenic vein.

SVT is a clinically relevant complication of acute pancreatitis, and it is imperative to delineate the management strategy for these patients. Although several complications other than SVT may arise in patients with acute pancreatitis, it is important to differentiate between patients that only require a standard approach to care and those that require advanced medical management. The strategies have ranged from conservative management, such as treating acute pancreatitis, to bowel rest, anticoagulation, thrombolytics, and even surgery [11]. Many researchers have proven CT imaging to be statistically significant in the accurate prediction of pancreatitis severity and prevalence of SVT [16].

This becomes especially helpful in determining whether conservative management is clinically useful in selective patients [16]. Standard management also entails critical monitoring of the disease for complications that may develop during hospitalization [16]. For example, SVT can increase venous pressure, which gives rise to portal hypertension and even further complications such as variceal bleeding [11]. This imposes a challenge in managing these patients, and whether to provide anticoagulants or not depends on the risk of gastrointestinal bleeding [11]. Additionally, some reports have determined splenomegaly as an asymptomatic sign of SVT that may require splenectomy before pancreatic surgery [16]. Based on the patient’s diagnostic tests, including imaging and laboratory results in the ED, she initially received conservative management with the administration of antiemetics, H2 blockers, IV opioids, and IV broad-spectrum antibiotics to address and manage her presenting symptoms on admission to the floor. Ultimately, the patient showed minimal improvement, and surgery was consulted for acute pancreatitis. A repeat CT scan with contrast allowed for critical monitoring of the disease and showed worsening pancreatitis and splenic vein with two nonocclusive thrombi.

Overall, there are many treatment options and ways to manage patients with SVT. A CT scan is useful in defining the anatomy for diagnosis and treatment planning, as well as for the etiology of portal hypertension [17]. Asymptomatic patients do not require further intervention [17]. While medical intervention for SVT includes anticoagulation to maintain an international normalized ratio (INR) between two and three [17], anticoagulation treatment with Lovenox was started to alleviate our patient’s symptoms and prevent the possible recurrence of thrombosis. Routine anti-factor Xa levels were evaluated roughly four hours after the initial Lovenox dosage to provide optimal dosage and then were repeated throughout the treatment course. The goal of anticoagulation is to prevent the extension of the clot and to allow for recanalization so that intestinal infarction and portal hypertension do not develop [16]. Hematology was consulted to further optimize the treatment plan for our patient, in which restarting Lovenox was suggested and to be continued daily for one month.

Patients with SVT secondary to acute pancreatitis can expect an overall good prognosis when treatment is started early or if the patient is asymptomatic. A misdiagnosis and or a delay in diagnosis and management may lead to a poor outcome. Observation of patients with pancreatitis-induced SVT is associated with an overall incidence of gastric variceal hemorrhage of approximately 4%, with no deaths secondary to variceal bleeding [7]. Varices demonstrated on CT are associated with a 5% risk of bleeding, but the presence of varices on endoscopy increases this risk [7]. Additionally, observation of SVT does not negatively affect the quality of life compared to patients who undergo surgical treatment for chronic pancreatitis [7]. Routine splenectomy for pancreatitis-induced SVT is not indicated given the low incidence of gastric variceal hemorrhage and the absence of mortality related to variceal hemorrhage [7]. Patients with SVT, particularly those with varices seen on CT, should undergo regular upper gastrointestinal endoscopy. Splenectomy should be reserved for those who experience bleeding or have an increased risk of hemorrhage. Given our patient’s early diagnostic and treatment intervention, her successful recovery from cholecystectomy, compliance with anticoagulation therapy, and monthly outpatient follow-ups with hematology, she continues to be monitored under the care of her primary physician and hematologist and is showing excellent improvement.

## Conclusions

Pancreatitis rarely presents in the pediatric population, especially when associated with vascular complications such as SVT. There is a lack of reported and studied cases for this particular presentation among the pediatric population, which makes it challenging to evaluate and compare information thoroughly. SVT is a clinically relevant complication of acute pancreatitis, so it is important to delineate the management strategy for these patients. Overall, there are many treatment options and ways to manage patients with SVT, and patients can expect an overall good prognosis when diagnosis and treatment are done early. As cases of acute pancreatitis rise in the pediatric population, there is a need for further evaluation of the incidence, presentation, and management strategies to deliver optimal care. The majority of literature and studies analyzing acute pancreatitis and SVT in the adult population places further significance on the need for exploration, specifically within pediatric cases presenting with SVT. This will further improve our understanding of this complication and provide optimal management.
